# Minimally invasive pouch technique with leukocyte platelet rich fibrin compared to non-invasive hyaluronic acid injection in reconstruction of interdental papilla in esthetic zone: a randomized clinical trial

**DOI:** 10.1186/s12903-025-06127-7

**Published:** 2025-07-26

**Authors:** Aya Alleithy, Hala Abuel-Ela, Susan Sarhan

**Affiliations:** 1 Department of Oral Medicine, Periodontology, and Oral Diagnosis, Faculty of Dentistry, Ain Shams University, Cairo, Egypt; 2 Department of Oral Medicine, Periodontology, and Oral Diagnosis, Faculty of Dentistry, Ain Shams University and Misr International University, Cairo, Egypt

**Keywords:** Papilla reconstruction, Platelet-rich fibrin, Hyaluronic acid gel, Pouch technique, Gingival black triangle

## Abstract

**Background:**

Interdental papilla deficiency is of high concern from the esthetic and functional perspectives. Several invasive approaches have been reported for interdental papilla reconstruction. However, the long-term success of invasive approaches is still controversial, therefore this study aimed to assess the minimally invasive Pouch technique using Leukocyte Platelet Rich Fibrin compared to non-invasive Hyaluronic acid gel injection in the reconstruction of Interdental papilla in esthetic zone.

**Methods:**

The study was designed as a randomized, controlled, double-blinded, and phase (IV) clinical trial. The trial was registered retrospectively with the date (21/7/2023) and registration number (NCT05953896). A total sample size of 20 cases was planned to be recruited. i.e. (10 patients in each group). Patients with deficient interdental papilla class I or II in the esthetic zone were selected, then patients were randomized using computer-generated block randomization into two equal groups: group A (L-PRF) and group B (HA gel). Blinding was performed in selection of patient intervention, while blinding of the intervention wasn’t applicable since both interventions are completely different. The clinical and radiographic parameters were assessed at 3 and 6 months, As well as evaluation of patient satisfaction between the two tested groups.

**Results:**

Both groups showed significant improvement in all clinical parameters at 3 months and 6 months postoperatively. Assessment of patient satisfaction showed overall improvement in both groups after 6 months, with successful reduction in the mean value of black triangle height of the PRF group: (0.9 ± 0.52), and HA group: (0.6 ± 0.46), with no significant difference** (***P value*: 0.178 ^ns^) between the two groups at 6 months. No significant difference was found in the primary outcomes, and secondary outcomes between the study groups at 6 months.

**Conclusions:**

Both injectable HA and multilayered L-PRF gave successful results that can be comparable to the invasive surgical techniques.

**Supplementary Information:**

The online version contains supplementary material available at 10.1186/s12903-025-06127-7.

## Introduction

Interdental papilla (IDP) is the part of the gingiva that occupies the contact area between teeth and supported by the interproximal bone. Reduced interdental papilla height can result in esthetic problems, it’s generally called the gingival black triangle or the papillary deficiency [1]. Chronic gingival inflammation, interproximal bone resorption, and periodontal disease are frequent causes for the loss of interdental papilla [2–4].

Tarnow et al. conducted a clinical study to evaluate the vertical distance between the contact point and the alveolar bone crest, it concluded that the presence or absence of the interdental papilla is determined by the distance between the alveolar crest and the apical part of the contact point between two teeth. The study results showed that when the measurement from the contact point to the alveolar crest of bone was 5 mm or less, the papilla was present almost 100% of the time. When the distance was 6 mm, the papilla was present 56% of the time, and when the distance was 7 mm or more, the papilla was present 27% of the time or less. For every millimeter increase, the chance of papilla presence reduced considerably [2].

Several surgical techniques have been reported; they are mostly invasive and unpredictable. Moreover, the success of surgical papilla regeneration depends on the gingival phenotype. Several minimally invasive techniques have been developed to reconstruct the deficient interdental papilla. The sub-epithelial connective tissue graft is believed to be the gold standard material for interdental papilla reconstruction, with long-term stable results and high predictability [3, 4].

Azzi et al. reported good results in previous case reports where patients subjected to surgical approach to restore the deficient papilla using a sulcular incision and a connective tissue graft placed under the buccal and palatal flap to optimize the blood supply to the grafted tissue [5]. This technique might have shown a better improvement in papillary height that could be due to the rich blood supply provided to the graft from both the buccal and palatal flaps.

Choukroun has introduced platelet-rich fibrin (PRF), a dense fibrin scaffold with a controlled slow release of multiple growth factors and glycoproteins [6]. With a surgical technique using multilayered (L-PRF) or an injection technique using (I-PRF), their biological properties help to maintain flap stability, enhance neo-angiogenesis, provide maximum coverage to the gingival flap, and promote the healing process. Platelet cytokines like platelet-derived growth factor (PDGF), transforming growth factor-beta (TGF-β), and insulin-like growth factor-1 (IGF-1) are gradually released as the fibrin matrix is resorbed [7].

Hyaluronic acid (HA) is a natural polysaccharide of the extracellular matrix of connective tissue of the dermis and gingiva, Synovial fluid, and other tissues. HA is effective for multiple oral applications because it participates in various biological processes like cell adhesion, differentiation proliferation, and cell signaling [8].

Therefore, this study was conducted to assess:

- The influence of hyaluronic acid gel injection versus multilayer leukocyte platelet rich fibrin on the height of the black triangle; the distance between the deficient papilla tip and the contact point (PT-CP distance) as the primary outcome.
- Patients’ satisfaction of their final esthetic appearance was assessed as the secondary outcome.

### Hypothesis

The study hypothesized that there is no significant difference between both techniques.

## Materials and methods

### Sample size

A total sample size of at least 20 cases was planned to be recruited. i.e. (10) for each group. Sample size calculation was performed using (G* power version 3.1.9.4) [9]. Power analysis was designed to have adequate power to apply a 2-sided statistical test of the research hypothesis that there is no difference between groups.

### Study design

This study was designed as a randomized, controlled, double-blinded, and phase (IV) clinical trial. The study is considered a Phase IV clinical trial since both materials are FDA approved and the study was intended to monitor their safety and measuring their effect on the height of the black triangle and patient satisfaction.

### Patient selection

The study was conducted on 20 patients recruited from the outpatient clinic of the Oral Medicine, Periodontology, and Oral Diagnosis department, Faculty of Dentistry, Ain Shams University. All new patients of the outpatient clinic were examined by clinic staff and those who met the eligibility criteria were asked to sign a consent after explaining the details of the study and then started the randomization process. The research ethics committee has approved the study (ID: IM012112) and was registered retrospectively with the clinical trial registration number (NCT05953896).

### Randomization and blinding

The study was designed as a randomized clinical prospective study in which eligible patients were randomized for assigned interventions in a 1:1 ratio into two groups (10 patients in each group) using computer-generated block randomization.^1^ Each allocation was enclosed in an opaque envelope and provided with a code. A trial-independent person was in charge of maintaining the envelopes and only unfolding them after the intervention. All measurements were performed by a single examiner. Also, the outcome assessor was blinded to the type of intervention. Blinding was performed in selection of patient intervention, while blinding of the intervention wasn’t applicable since both interventions are completely different. The analysis approach was specified as per-protocol analysis and included only participants who adhered to the study protocol and completed the study according to the specified guidelines. There were no modifications added to the protocol after trial commencement. Also there were no changes of outcomes after the beginning of trial. The trial ended as per protocol after 6-month follow-up.

Patients were selected based on the following criteria: The patient's age ranged from 18 to 45 years. Systemically healthy patients. Patients with deficient IDP class I or II in the esthetic zone of upper and lower anterior and premolar teeth according to [10] Radiographic evidence of sufficient interdental alveolar bone (i.e., the vertical distance from the interdental contact point to the alveolar bone crest is ≥ 5 mm) [2]. A thick periodontal phenotype is present in the area to be treated (≥ 1 mm), the gingival thickness at the tested sites was measured using a graduated UNC-15 periodontal probe.^2^ Patients have good oral hygiene and high esthetic demand and are concerned about management of "black triangles."

Patients were excluded from the study considering the following criteria: Teeth with periapical infections, high frenum attachment at the site of deficient papilla. patients’ incompliance to treatment and persistence of gingival inflammation after phase I therapy, pregnant and lactating female patients, patients with para-functional habits or local factors such as teeth malocclusion and interdental spacing, smokers, alcoholics or drug abusers, and vulnerable groups of patients.

All patients had deficient papillae adjacent to teeth (11 sites with class I papilla deficiency and 9 sites with class II papilla deficiency based on Nordland and Tarnow’s classification [10]. Group A (Multilayer-L-PRF) included 10 patients who received interdental papillary reconstruction using multiple layers of L-PRF. Layers of L-PRF membranes were placed in each patient according to the compressibility of the tissues (three layers) as shown in figure (S1). Group B (injection with Hyaluronic acid gel): included 10 patients who received interdental papillary reconstruction using FDA approved Hyaluronic acid gel.^3^ Interim analysis was done at 3 months and 6 months (clinical and radiographic evaluation of effectiveness of the planned intervention and evaluation of safety concerns that might require modifications).

**Fig. 1:**
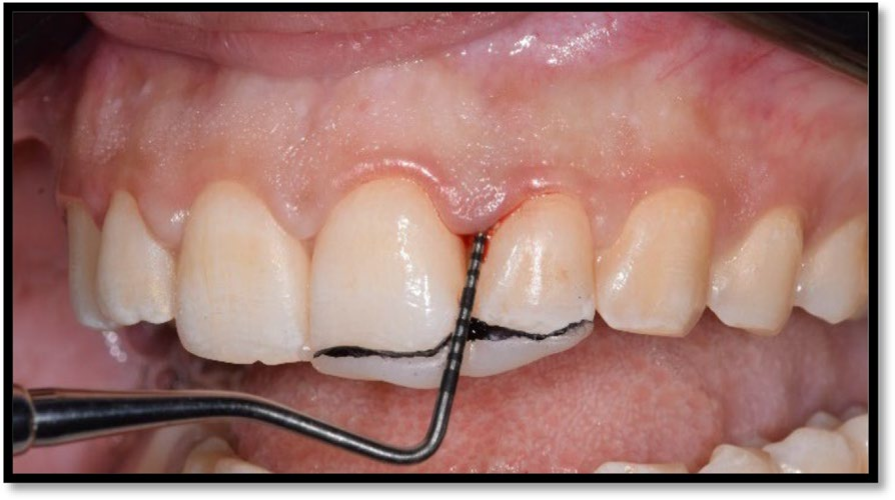
Composite stent used as a reference point for a standardized clinical measurement

The degree of papillary deficiency was assessed according to Nordland and Tarnow’s (1998) classification [10]. Initial periodontal therapy including full mouth supra-gingival scaling and sub-gingival debridement were performed with patient education for proper oral hygiene care. Patients’ re-evaluation for the maintenance oral hygiene care and reassessment of gingival condition was done four weeks after initial therapy. A modified stent was prepared on the day of the surgery and used for a standardized vertical probing for the clinical measurements of (PT-CP) distance, periodontal probing depth, and clinical attachment level [11]. The stent was made of light cured composite material with trimming of the interdental groove to allow the probe to touch the interdental papilla and allow calibration in a parallel direction to the long axis of the tooth as shown in Fig. 1.

All the clinical parameters were measured using a graduated UNC-15 periodontal probe. Standardized photographs were recorded following the photographic standardization protocol of a study reported by Abdelraouf et al. (2019); many variables were fixed every time photos were taken [12]. These variables included the patient's head position in the space, the Camera position in the space (relation between camera and patient), the camera's settings, and external flash settings.

## Assessment

### A-clinical assessment

The clinical outcomes were assessed three times for each patient; at baseline (preoperative), three months, and six months postoperatively and included the following measurements.

### Modified Papillary Bleeding Index (MPBI)

Was measured with a graduated UNC-15 periodontal probe, the appearance of bleeding was recorded with grades from 0 to 3 according to Barnett et al. (1980) bleeding index [13].

### Clinical attachment level (CAL)

According to Glavind and Löe, 1967 CAL was measured with the periodontal probe from CEJ to the bottom of the pocket to the nearest mm [14].

### Periodontal Probing Depth (PPD)

PPD was measured from the gingival margin to the bottom of the pocket to the nearest mm.

### The distance between the papilla tip and contact point (PT-CP)

The clinical distance from papilla tip to contact point was measured at the sites of papillary deficiency using a graduated UNC-15 probe and a customized composite stent, also clinical photograph was taken using reproducible alignment device and endodontic ruler for reference to papilla height.

### Surface area of black triangle (SABT)

Pre-operative and postoperative photographs at three months and six months’ intervals were taken perpendicular to teeth of interest. Reproducible alignment device was used. The device was used to take subsequent photos as close to the original photos as possible. Then the surface area of the black triangle (SABT) was measured. These photos were analyzed using the ImageJ2 processing software.^4^ Program was used to measure the height of the selected papillae [15].

### B-radiographic assessment

**Fig. 2:**
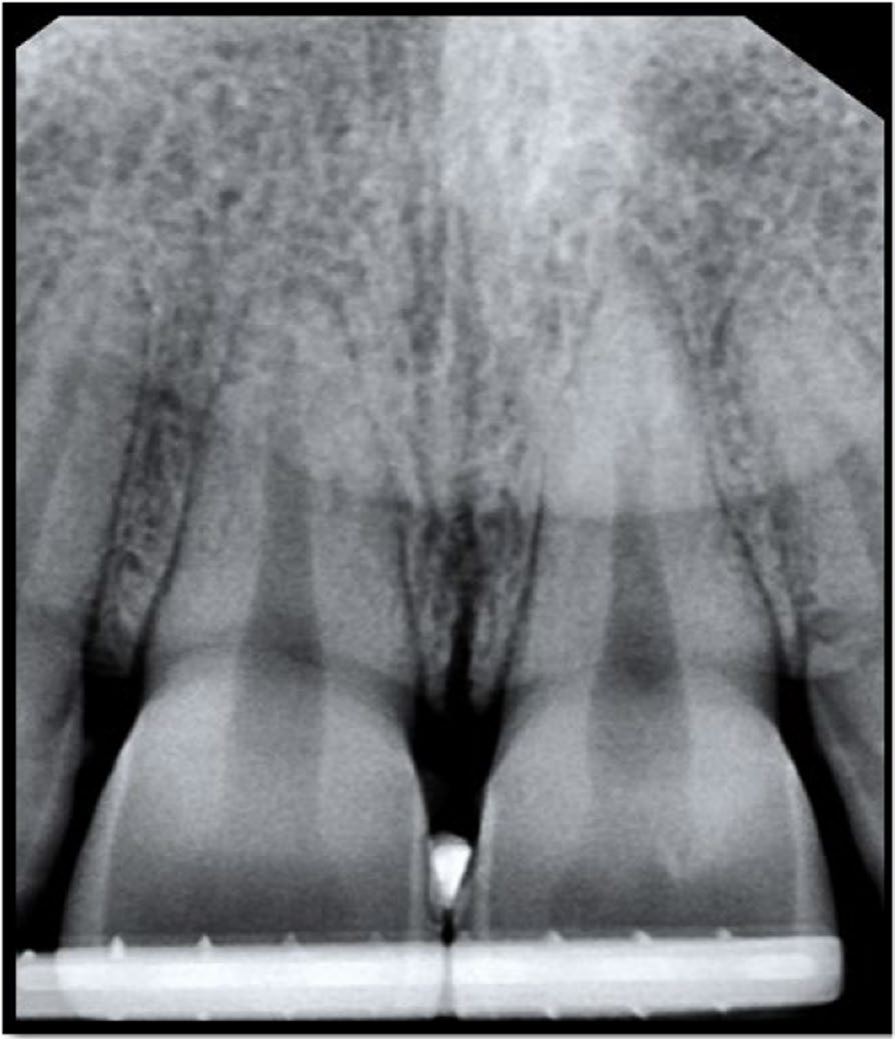
Digital periapical radiographs with a reproducible alignment device for standardization while radiopaque material placed at papilla tip

Radiographs were taken pre-operatively and postoperatively at three months and six months’ intervals [16]. The Digital periapical radiographs were taken using a reproducible alignment device for standardization while Metapex^5^ radiopaque material placed at papilla tip as showed in Fig. 2. The distance from the alveolar bone crest to the root apex (BC-RA) was measured and used as a reference to evaluate the change, if any, in the level of alveolar bone crest.

### C-assessment of patient satisfaction

Was recorded through evaluation of the following: The Pain-visual analog scale (P-VAS), The patient satisfaction questionnaire short-form (PSQ-18), and the global aesthetic improvement scale (GAIS).

### The pain-visual analog scale (P-VAS)

It was recorded postoperative or post injection and at each follow up period. The numerical rating scale for pain (NRS) developed by Downie in (1978) as described from 0 to 10 [17].

### The patient satisfaction questionnaire (short-form, PSQ-18)

Was recorded postoperative or post injection [18].

### The global aesthetic improvement scale (GAIS)

Was recorded at 3 and 6 months follow up periods [19].

## Surgical procedures

### Group A (Multilayer L-PRF)

**Fig. 3:**
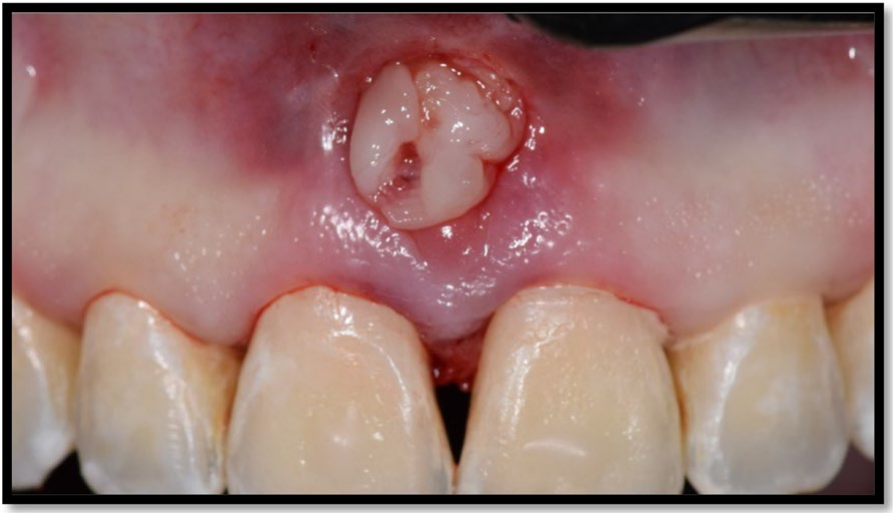
Placement of L-PRF membranes in the pouch created apical to the papilla and the (multilayer) L-PRF membranes were adjusted to fill the papilla

A single 3 mm semilunar incision has been made in the buccal vestibule, apical to the mucogingival junction in the mid-interproximal area of the papilla to be treated. Incisions were made around the interdental papilla. Intra-sulcular releasing incisions have been made on the teeth adjacent to the papilla to be augmented, extending from the buccal aspect to the interdental aspect of the papilla without altering the integrity of the papilla. Then, a curette was used around the necks of the involved teeth to facilitate the coronal displacement of the gingiva-papillary unit. A pouch was created by its continuation in the interdental area and extended to the distal margins of the adjacent teeth on both sides of the deficient papilla. Tunneling of the incisions maintained the full height and thickness of the gingiva. L-PRF has been prepared according to Pinto’s protocol immediately prior to the surgery, 10 ml of blood (for each membrane) was collected from the patient by venipuncture of antecubital vein and then drawn quickly into sterile glass tube without addition of anticoagulant [20]. The tubes were immediately centrifuged for 12 min at (400 g RCF) equivalent to 2700 rpm at room temperature by using the Intra-Spin centrifuge.^6^ The L-PRF clot formed in the centrifuged tube was then removed by sterile tweezers and placed in PRF box^7^ for gentle compression by gravity to be used after 5 min. The membranes were cut into pieces and the (multilayer) L-PRF was adjusted to fill the papilla as showed in Fig. 3. Then, the incision was closed with simple interrupted sutures,^8^ and the whole complex was secured using sling sutures placed on stops created on the teeth using composite. The sutures were removed after two weeks from surgery.

### Group B (HA injection)

**Fig. 4:**
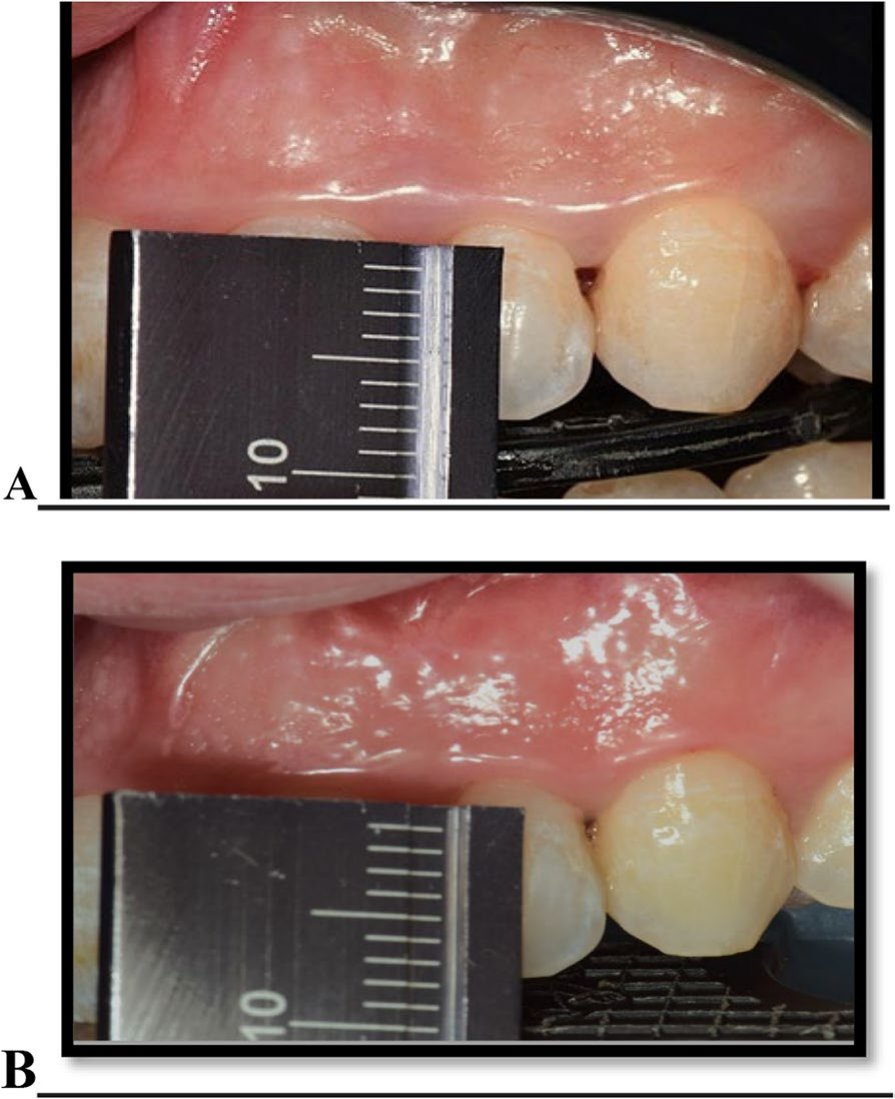
Clinical measurement of papilla-tip contact point distance at baseline (**A**) and 6 months with a ruler used as reference (**B**)

After administration of local anesthesia, a 30-gauge needle was used to inject 0.1 to 0.2 ml of the FDA-approved HYALGAN® gel with (2%) concentration of hyaluronic acid sodium salt in a pre-packed syringe as shown in figure (S2). The hyaluronic acid gel was injected 2-3 mm apically to the coronal tip of the involved papillae and directed coronally with an angulation of 45° to the long axis of the tooth until the papilla became blanched. Then, gauze lightly molded the papilla in an incisal direction for 1 min. The injection was applied at each papilla at the following intervals: baseline, one week, and two weeks [11]. The change in the IPD height from baseline (A) to 6 months (B) is shown in Fig. 4.

## Photographic analysis

**Fig. 5:**
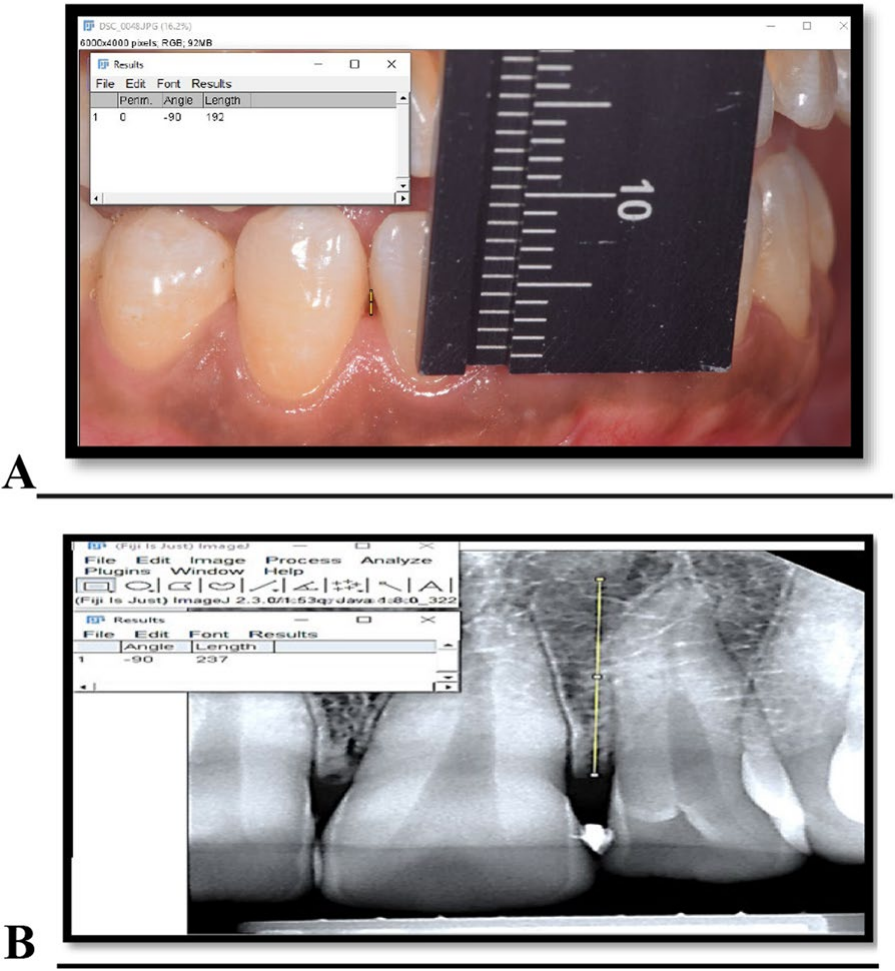
Photographic analysis (**A**) and Radiographic analysis (**B**)

The black triangle area was photographed using a Nikon d5300 DSLR camera with Nikon macro lens with fixed magnification, and all standardization methods were applied. We used ImageJ2 software.^9^ to verify the clinical data and measure the change in black triangle height through the six-month follow-up period as illustrated in Fig. 5. Pre-operative and postoperative photographs at three-month and six-month intervals were taken perpendicular to teeth of interest using a reproducible alignment device to take subsequent photos as close to the original photos as possible [15].

## Radiographic analysis

Digital periapical radiographs were taken using a reproducible alignment device for standardization, while Metapex^10^ Radiopaque material was placed at the papilla tip, and the distance from the alveolar bone crest to the root apex (BC-RA) was measured and used as a reference to evaluate the change, if any, in the level of the alveolar bone crest. Radiographs were taken pre-operatively and postoperatively at three-month and six-month intervals [16]. Then, the digital radiographs were analyzed, and the (BC-RA) distance was measured using the ImageJ2 software as illustrated in Fig. 5.

### Statistical analysis

Sample size calculation was performed using (G* power version 3.1.9.4), based on previous studies results [21, 22], which showed a significant difference in the improvement of papillary height between the control and test group with an expected effect size of approximately 2.137. A total sample size of 20 (10 patients in each group) after adjustment for non-parametric test usage and losses of follow up will be sufficient to detect such an effect size using a power of 90%, and a significance level of 5%. Data management and statistical analysis were performed using the Statistical Package for Social Sciences software. Numerical data were summarized using mean, standard deviation, median and range. Data were explored for normality by checking the data distribution using Shapiro–Wilk test. Comparisons between groups with respect to normally distributed numeric variables were performed using independent t test. Comparison between different observations was performed using repeated measures ANOVA test. Comparisons between groups with respect to non-parametric numeric variables were performed using Mann Whitney U test. Comparison between different observations was performed using Friedman test and Wilcoxon signed rank test. The percent change was calculated by the formula: (Value after-value before)/value before × 100. Qualitative data were expressed as number and percentage. Chi square test was used intra and intergroup for comparisons. All *p*-values are two-sided. *P*-values ≤ 0.05 were considered significant.

## Results

**Fig. 6:**
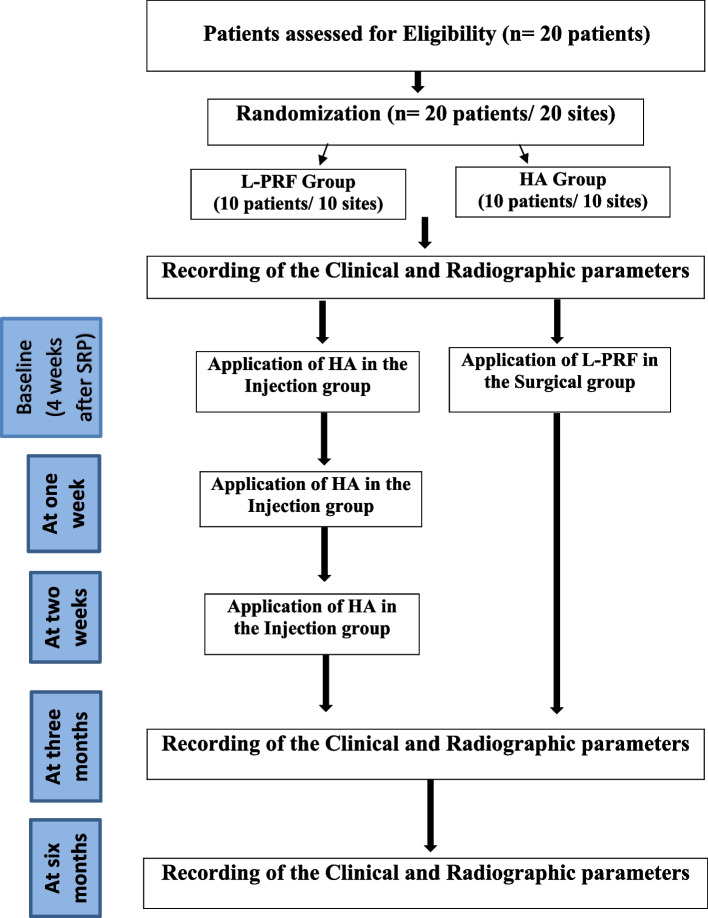
Flowchart of the selection process, treatment and follow-up of patients

The study was conducted on 20 cases that were randomly and equally allocated to two studied groups. There were no dropouts or missing data in the study. Figure 6 shows a Consort flowchart for recruitment, treatment and follow-up of patients.

### Clinical and radiographic parameters

**Fig. 7:**
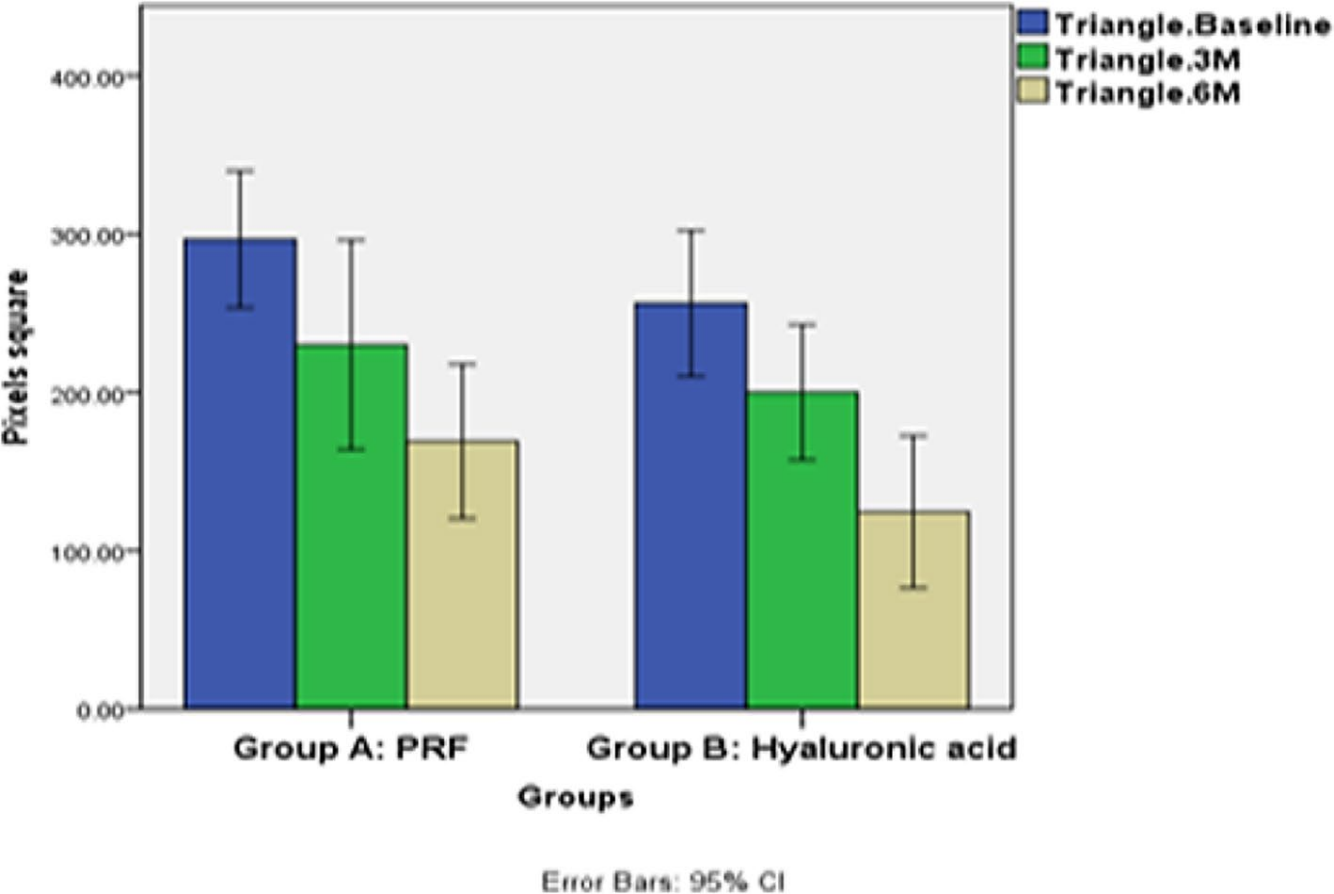
Bar chart illustrating mean (SABT) in Group A and group B at baseline and after 3 and 6 months

The clinical and radiographic parameters for the (L-PRF) and (HA) groups are represented in Table 1 as descriptive statistics. For the parameters of (PT-CP), (SABT), and (BC-RA), both groups showed a decrease in the mean value of the parameters, with no significant difference after 6 months (Fig. 7).

**Table 1:** Descriptive statistics of clinical and radiographic parameters between the study groups (Intergroup comparison) and the changes by time within each group (Intragroup comparison)

Parameters/Groups	PRF Group (Mean ± SD)	HA Group (Mean ± SD)	*p*-value (intergroup)	Cohen’s d (95% CI)	Mean Difference	(95% CI) of the Difference
PT-CP (mm)						
Baseline	2.55 ± 0.75	1.95 ± 0.96	0.430 ^ns^	0.70 (−0.22 to 1.59)	0.65	(−0.48 to 1.78)
3 months	1.5 ± 0.75	1.15 ± 0.71	0.231^ns^	0.48 (−0.42 to 1.36)	0.75	(−0.07 to 1.57)
6 months	0.9 ± 0.52	0.6 ± 0.46	0.178^ns^	0.61 (−0.30 to 1.50)	0.65	(−0.05 to 1.35)
*p*-value (intragroup)	0.000*	0.000*				
Mean difference (from baseline to 6 months)	−1.35 ±.34	−1.35 ±.75	0.634 ^ns^	0.00 (−0.62 to 0.62)	0.00	(−0.59 to 0.59)
Percentage change (%) (from baseline to 6 months)	−63.5 ± 15.76	−70 ± 20.49	0.528 ^ns^	0.36 (−0.27 to 0.98)	11.56	(−6.39 to 29.51)
SABT (square pixels)				0.65 (0.01 to 1.28)		
Baseline	296.6 ± 60.32	256.1 ± 64.18	0.163 ^ns^		40.50	(−18.01 to 99.01)
					30.00	(−43.03 to 103.03)
					44.50	(−19.04 to 108.04)
3 months	230 ± 92.35	200 ± 59.63	0.399^ns^	0.39 (−0.24 to 1.01)		
6 months	169 ± 68.06	124.5 ± 67.2	0.158^ns^	0.66 (0.02 to 1.29)		
*p*-value (intragroup)	0.000*	0.000*				
Mean difference (from baseline to 6 months)	−127.6 ± 30.24	−131.6 ± 58.97	0.910 ns	0.09 (−0.54 to 0.70)	4.00	(−41.12 to 49.12)
Percentage change (%) (from baseline to 6 months)	−45.46 ± 20.11	−52.32 ± 26.52	0.449 ns	0.29 (−0.33 to 0.91)	6.85	(−15.37 to 29.08)
BC-RA (pixels)						
Baseline	180.80 ± 9.76	182.70 ± 6.15	0.609 ns	−0.23 (−0.85 to 0.39)	−1.90	(−9.56 to 5.76)
3 months	179.50 ± 9.78	182.70 ± 6.15	0.392 ns	−0.39 (−1.02 to 0.24)	−3.20	(−10.87 to 4.47)
6 months	178.30 ± 9.66	182.70 ± 6.15	0.240 ns	−0.54 (−1.17 to 0.09)	−4.40	(−12.00 to 3.20)
*p*-value (intragroup)	.85 ^ns^	1 ns				
Mean difference (from baseline to 6 months)	−2.5 ± 1.43	0.00 ± 0.00	0.000*	−2.47 (−3.29 to −1.63)	−2.50	(−3.45 to −1.54)
Percentage change (%)(from baseline to 6 months)	−1.38 ±.8	0.00 ± 0.00	0.000*	−2.44 (−3.26 to −1.60)	−1.38	(−1.90 to −0.85)

^*^significant (*p* ≤ 0.05), ns; non-significant (*p* > 0.05)

The clinical parameters (CAL), (PPD), and (MPBI) are represented in Table 2 as descriptive statistics. The mean value of the parameters significantly reduced after 6 months, with no significant difference between the two groups.

**Table 2:** Descriptive statistics of clinical periodontal parameters between the study groups (Intergroup comparison) and the changes by time within each group (Intragroup comparison)

Parameters/Groups	PRF Group (Mean ± SD)	HA Group (Mean ± SD)	*p*-value (intergroup)	Cohen’s d (95% CI)	Mean Difference	(95% CI) of the Difference
MPBI						
Baseline	0.50±0.53	0.60±0.52	0.661 ^ns^	-0.19 (-0.81 to 0.43)	-0.10	(-0.59 to 0.39)
3 months	0.40±0.52	0.20±0.42	0.342 ^ns^	0.42 (-0.21 to 1.05)	0.20	(-0.24 to 0.64)
6 months	0±0	0±0	1 ^ns^	NA	0.30	(-0.10 to 0.70)
* p*-value (intragroup)	0.015*	0.009*				
Mean difference (from baseline to 6 months)	-0.50±0.53	-0.60±0.52	0.661 ^ns^	0.19 (-0.43 to 0.81)	0.10	(-0.39 to 0.59)
Percentage change (%) (from baseline to 6 months)	-50.0±52.7	-0.60±51.64	0.661 ^ns^	-0.95 (-1.60 to -0.29)	10.0	(-0.39 to 0.59)
CAL (mm)						
Baseline	0.80±0.92	0.60±0.84	0.615 ^ns^	0.23 (-0.40 to 0.85)	0.20	(-0.62 to 1.02)
3 months	0.35±0.58	0±0	0.068 ^ns^	0.85 (0.20 to 1.50)	0.35	(-0.03 to 0.73)
6 months	0±0	0±0	1 ^ns^	NA		(-0.06 to 0.76)
* p*-value (intragroup)	0.009*	0.018*			0.10	
Mean difference (from baseline to 6 months)	-0.8±.92	-.60±.84	0.615 ^ns^	-0.23 (-0.85 to 0.40)	-0.20	(-1.02 to 0.62)
Percentage change (%) (from baseline to 6 months)	-50±52.7	-40±51.64	0.661 ^ns^	-0.19 (-0.81 to 0.43)	-0.20	(-59.02 to 39.02)
PPD (mm)						
Baseline	2.60±1.41	2.30±1.30	0.585 ^ns^	0.22 (-0.40 to 0.84)	0.30	(-0.97 to 1.57)
3 months	1.85±0.97	1.50±0.75	0.372 ^ns^	0.40 (-0.23 to 1.03)	0.35	(-0.46 to 1.16
6 months	1.35±0.63	1.25±0.42	0.797 ^ns^	0.19 (-0.44 to 0.81)	0.10	(-0.40 to 0.60)
* p*-value (intragroup)	0.002*	0.001*				
Mean difference (from baseline to 6 months)	-1.25±1.11	-1.05±.98	0.585 ^ns^	-0.19 (-0.81 to 0.43)	-10.00	(-1.18 to 0.78)
Percentage change (%) (from baseline to 6 months)	-38.39±28.66	-34.52±26.18	0.695 ^ns^	-0.14 (-0.76 to 0.48)	-3.86	(-29.66 to 21.92)

*; significant (*p* ≤ 0.05), ns; non-significant (*p*>0.05)

#### Patient satisfaction parameters

**Fig. 8:**
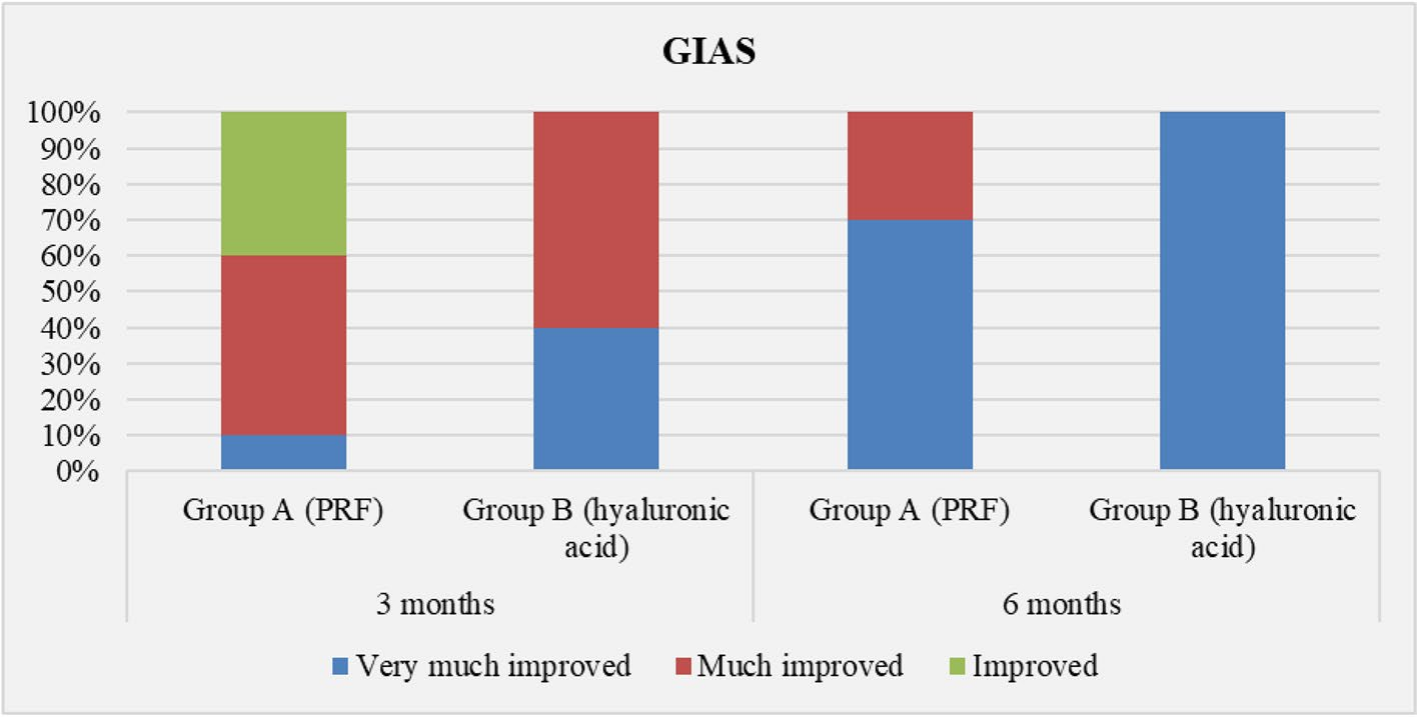
Frequency of GAIS scores in group A (L-PRF) and group B (HA) at baseline, 3 and 6 months

**Fig. 9:**
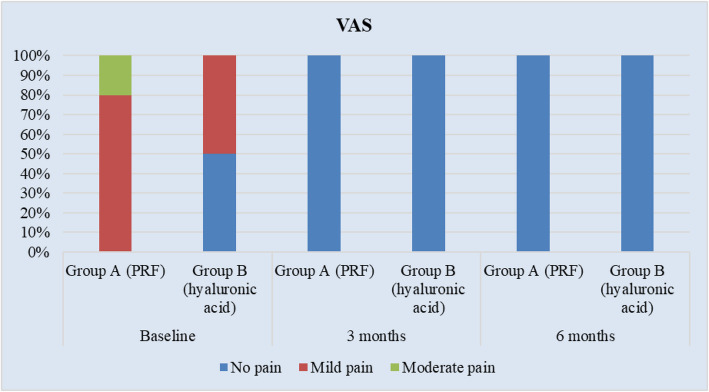
Frequency of VAS scores in (L-PRF) group and (HA) group at baseline, 3 and 6 months

Data are represented in Table 3 as descriptive statistics; assessment of (PSQ-18) showed no significant difference between the two groups. The intragroup comparison for (GAIS) showed overall improvement in both the study groups at 6 months, while the intergroup comparison showed that the difference between groups was not statistically significant at 6 months, the frequency of GAIS scores in group A (L-PRF) and group B (HA) is illustrated in Fig. 8. The intragroup comparison of (VAS) showed a significant difference after 6 months, while the intergroup comparison showed a significant difference at baseline. However, there was no significant difference between groups at 6 months, the frequency of (VAS) scores in (L-PRF) group and (HA) group at baseline, 3 and 6 months is illustrated in Fig. 9.

**Table 3:** Descriptive statistics of patients’ satisfaction scores between the study groups (Intergroup comparison) and the changes by time within each group (Intragroup comparison)

Parameters/Groups	PRF Group (Mean ± SD)	HA Group (Mean ± SD)		*p*-value (intergroup)			Mean Difference	95% Confidence Interval of the Difference
PSQ-18								
Total score	51.90±.57	51.40±2.01.459^ns^ Cohen’s d=0.34 (-0.29 to 0.96) 0.31 (-0.31 to 0.93)					0.50	(-0.88to 1.88)
Average score	3.24±0.04	3.21±0.13				.459 ^ns^	0.03	(-0.056 to 0.118)
(GAIS)		PRF Group		HA Group				
		No.	%	No.	%	*p*-value (intergroup)		
3 months	Very much improved	1	10	4	40	0.053 ^ns^ Cohen’s Omega =0.543 (0.000:0.946)		
	Much improved	5	50	6	60			
	Improved	4	40	0	0			
6 months	Very much improved	7	70	10	100	0.06 ^ns^ Cohen’s Omega =0.420 (0.000:0.858)		
	Much improved	3	30	0	0			
*p*-value(intragroup)	0.011*					0.003*		
(VAS)								
		Group A (PRF)				Group B (HA)		*p*- value (intergroup)
		No.	%			No.	%	
Baseline	No pain	--	--			5	50	0.021* [Cohen’s omega=0.620 (0.066:1.000)]
	Mild pain	8	80			5	50	
	Moderate pain	2	20			---	---	
3 months	No pain	10	100			10	100	1 ^ns^ [NA]
6 months	No pain	10	100			10	100	1 ^ns^ [NA]
*p*-value (intragroup)		0.00005*				0.036*		1 ^ns^ [NA]

*; significant (*p* ≤ 0.05), ns; non-significant (*p*>0.05)

No significant difference was found in the primary outcomes, such as (PT-CP), (SABT), (BC-RA), and secondary outcomes (patient satisfaction) between the study groups at 6 months.

## Discussion

The interdental papilla is the part of the gingiva that occupies the gingival embrasure at the contact area between teeth. The position and shape of the interdental papilla are based on the underlying alveolar bone, the level of contact point, and teeth position. A deficient interdental papilla can cause food impaction and esthetic problems. Over the years, several surgical and nonsurgical techniques have been developed to reconstruct the lost papilla [23].

Connective tissue graft is believed to be the gold standard matrix for the augmentation of IDP, the inherent regenerative potential of CTG helps to improve the reconstruction technique. PRF also contributes to the augmentation of the deficient papilla, the efficacy of PRF is slightly lower in restoring papilla height and not comparable to CTG. However, many studies have reported no significant difference between the CTG and PRF groups when the gain of papilla height was evaluated after 6 months [24].

In contrary to our study that used PRF in the surgical reconstruction of IDP, the use of sub-epithelial connective tissue graft is still believed to be the gold standard for periodontal plastic surgeries including papilla reconstruction, with long-term stable results [25].

PRF is a dense fibrin scaffold with a controlled release of glycoproteins and growth factors (TGF-β, PDGF-AB, and VEGF). On this basis, we used PRF in our study since it enhances the potential of tissue regeneration by induction of migration and proliferation of human mesenchymal stem cells (MSCs). Also PRF has a great anti-inflammatory and anti-microbial activity, that contribute to better tissue healing and regeneration [26].

The combination of PRF and the pouch technique was reported by Sharanappa et al., [27]. Our study used this pouch-like design as it eliminates excessive trauma to the soft tissues, contributes to the dual blood supply of the tissues and maintains the flap in a stable position. Moreover, it avoids incisions interrupting the vascular supply at the papillary area and allows for esthetic appearance of restored papilla with longtime stable results.

Researchers considered HA concentration as an important factor in the management of interdental papilla deficiency. A study reported by Tanwar and Hungund (2016) that used HA gel in three concentrations, 1%, 2%, and 5%, to evaluate its efficiency in interdental papilla reconstruction; 5% of HA has given highly significant results [28]. Researchers concluded that 5% HA is effective for regeneration of deficient interdental papillae with the slightest rebound after six months. Similarly, in the current study, injection with the approved drug HYALGAN® (2%) resulted in successful papillary reconstruction with improvement in the assessed clinical parameters and stable results after 3 and 6 months of injection.

In contrary to our study that used unidisciplinary approach for IDP reconstruction, several studies have reported successful papillary reconstruction and maximum esthetic results by multidisciplinary approaches. Previous case reports by Carnio and Carnio (2018) demonstrated (periodontal, orthodontic, and restorative approaches) to reconstruct severe papilla deficiency in the esthetic zone with the association of different specialties [29]. Successful results were obtained at the 2-year follow up, after the perio-ortho phase was finished. The cooperation between periodontics-orthodontics and restorative specialists has a great importance in achieving successful results especially in severe cases of papillary deficiency.

Several protocols of HA injection are reported in the literature for IDP regeneration, regarding the number of injections, there is no consensus in the works studied, although the minimum number of injections was three. Generally, the treatment is carried out as follows: in each session, a single dose of 0.1–0.2 ml HA was used with a maximum of five applications or until black triangles were no longer visible. The time interval between applications was 3 weeks. The HA injection technique is effective for the regeneration of IDP defects within 6 months of injection. HA injection has the potential benefits of being efficient, predictable, and non-invasive. The main limitation of the technique is the maximum increase of papilla height that can be achieved, which needs to be maintained with subsequent injections. However, more clinical studies are recommended to approve the long term results and possibility of relapse after injections [30].

Several studies have demonstrated that HA is an effective method to promote regeneration of deficient interdental papillae. Another study by Ni et al., (2021) reported that the injection of HA could increase the papilla height and reduce the area of the black triangles, the same HA product significantly enhanced the migration and proliferation of the gingival fibroblasts [31]. Due to its high hydrophilicity, HA forms hydrogen bonds with nearby carboxyl and N acetyl groups when it comes into contact with water, allowing it to hold onto water, concentrate it into large concentrations, occupy a large volume in relation to its mass, and withstand compression, this phenomenon explains the successful results of HA injection for facial and gingival re-volumization [32].

HA is found most abundantly in the extracellular matrix of connective tissues. Synthesized HA is directly secreted into the extracellular space. It is also produced by fibroblasts in the presence of endotoxins. It is involved in tissue repair and wound healing by stimulating cell proliferation, migration and angiogenesis. Also, it has a major role in space-filling owing to its hygroscopic nature. Moreover, it regulates osmotic pressure and helps in maintaining the structural and homeostatic integrity of tissues [33].

The present study used an injection protocol with number of three injections at (baseline-one week- two weeks) intervals in similarity to the injection protocol reported in the study by Singh and Vandana (2019), moreover the same study has compared the effect of different concentrations of HA on IDP reconstruction and the results showed that the use of HA is effective for treatment of IDP loss [11].

The timing for follow up at 3 months and 6 months was decided based on several clinical studies on IDP using different reconstruction techniques. Favorable results were achieved and were stable at 6 months follow-up time [4].

In the current study there was a significant reduction in PT-CP distance in both groups which reflects the positive results of the non-invasive injection of HA in regeneration of deficient interdental papilla very similarly to the surgical group without the need for surgical intervention. These results remained stable through the follow-up period and were in contrast to what was reported by Aspalli et al., (2015) when they conducted their case report for treatment of deficient interdental papilla with failure of papilla regeneration after six months of intervention, this may be attributed to the limitations of surgical approach within the interdental papilla region [30].

The results of the present study were in accordance with a previous study by Ahila et al., (2018) who reported that the mean of PT-CP distance has decreased significantly after 3 months and 6 months following treatment with PRF, they concluded that the use of PRF achieved successful and predictable results in the management of papillary deficiency [22].

In accordance with results of the present study, Becker et al., (2010) conducted a study using injectable HA gel to reconstruct the interdental papilla; they reported successful results following the injection of HA into small papillary deficiency between implants and teeth. Improvements were assessed for a range of 6 to 25 months and they reported stable results through the follow up period [34].

The current study resulted in a marked improvement in both groups regarding the gain of IDP height. These results agree with Mansouri et al., (2013) who demonstrated that application of HA gel was successful for reconstruction of interdental papilla in the maxillary esthetic zone at six months follow up [4].

On assessing patient satisfaction, L-PRF group had a significantly higher total mean value of pain score than the HA group at baseline, the difference between groups was statistically significant (*p* = 0.021). This was attributed to the difference between the minimally invasive and non-invasive techniques used in the present study. Patients who were subjected to surgical intervention felt more pain postoperatively compared to patients who were managed with HA injection. At 3 and 6 months, 100% of cases in both groups had no pain, with no significant difference between the two groups (*p* = 1).

When compared to surgical techniques which are less predictable and painful, nonsurgical techniques are preferred due to their cost effectiveness, less stressful and achieving immediate results with high satisfaction rate. Nonsurgical approaches include correction of traumatic oral hygiene procedure, restorative techniques, orthodontic movement, repeated scrapping of the papilla and tissue volumizers (I-PRF and HA gel injection) Shawky and Darwish (2017) and (Singh, 2019). Thus in the current study we used HA for the management of class I and class II papilla deficiency [11, 35].

The results of the present study showed that both procedures were efficient for class I and II papilla reconstruction (Tarnow’s classification) in the esthetic zone. This was in agreement with the reviews done by Zhang et al., (2020) and Mahmoud (2022) to evaluate the IDP reconstruction using different approaches, most of them involve surgical intervention and yield unpredictable results, while minimally invasive treatment had the advantages of being effective, predictable, and less traumatic as compared to surgical treatment with maximum patient satisfaction. These reviews showed that the clinical application of both HA and PRF has become popular and successful choice in restoration of lost interdental papillae [36, 37].

Among the limitations of the present study that follow up time appears short when compared to the follow up periods reported in other studies in the literature, longer follow up periods would allow to assess the stability of results overtime. Different HA injection protocols could be applied, also the application of different biomaterials could be added in the test groups to evaluate their effect on IDP reconstruction. Volumetric analysis was not used in the present study; it may be useful for more accurate assessment of results. This study has involved one center; for further validation of the results, a multi-centered study with larger sample size must be conducted. Additionally, different concentrations of HA gel and different forms of PRF can be applied in further studies.

## Conclusions

The present study shows a novel approach for using Hyaluronic acid injection to reconstruct mild and moderate interdental papillae loss. The L-PRF group represented the control group since its results are well documented in the literature compared to other treatment modalities. Further clinical trials using L-PRF in combination with connective tissue grafts to reconstruct deficient papillae are needed. Studies using other slowly degradable materials to augment interdental papillae are also recommended. Furthermore, studies with larger sample size and long-term follow-up to approve the stability of the results are required.

## Supplementary Information

Supplementary Material 1.

## Data Availability

The data that support the findings of this study are available from the corresponding author but restrictions apply to the availability of these data, which were used under license for the current study, and so are not publicly available. Data are however available from the authors upon reasonable request with permission of the corresponding author.

## Abbreviations

IDP: Interdental Papilla
PRF: Platelet rich fibrin
HA: Hyaluronic acid
CTG: Connective tissue graft
PPD: Periodontal Probing Depth
CAL: Clinical Attachment Level

## Footnotes

1 SPSS, version 22.0. IBM comp. Armonk, NY.

2 Nordent®, UNC-15. Germany.

3 HYALGAN (Sodium Hyaluronate), Fidia Pharma, USA Inc.

4 Image J2. The National Institute of Health-USA.

5 Metapex®, MetaBioMed comp., Korea.

6 IntraSpin™ Centrifuge; Intra-Lock International. USA.

7 Expression™ kit. USA.

8 Assut® Polypropylene, non-absorbable, 4/0 reverse cutting 3/8 circle 19 mm. Switzerland.

9 Image J2. The National Institute of Health-USA.

10 Metapex, MetaBioMed, Korea.
