# Supplementary material for: Minimally invasive pouch technique with leukocyte platelet rich fibrin compared to non-invasive hyaluronic acid injection in reconstruction of interdental papilla in esthetic zone: a randomized clinical trial
Source: BMC Oral Health. 2025 Jul 26;25:1265. doi: 10.1186/s12903-025-06127-7 (PMC12297722; doi:10.1186/s12903-025-06127-7)
Supplement: Supplementary file 1 — Supplementary Material 1. [file 12903_2025_6127_MOESM1_ESM.docx]

**
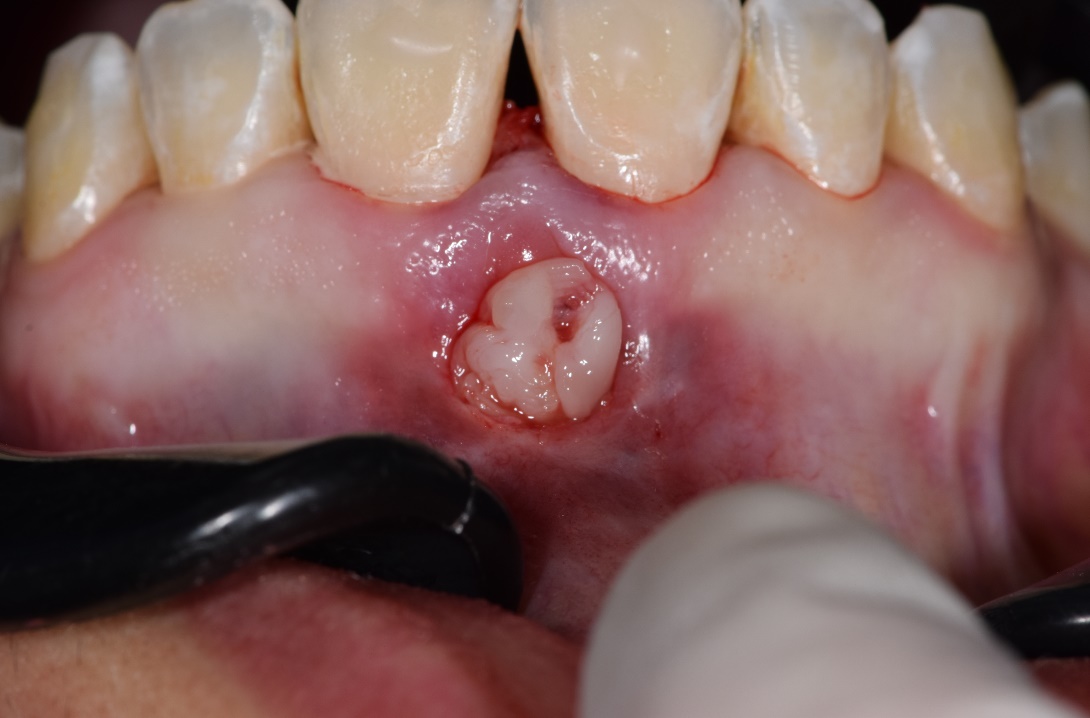
**

**Figure (S1): Placement of L-PRF membranes in the pouch created apical to the papilla and the (multilayer) L-PRF membranes were adjusted to fill the papilla.**

**
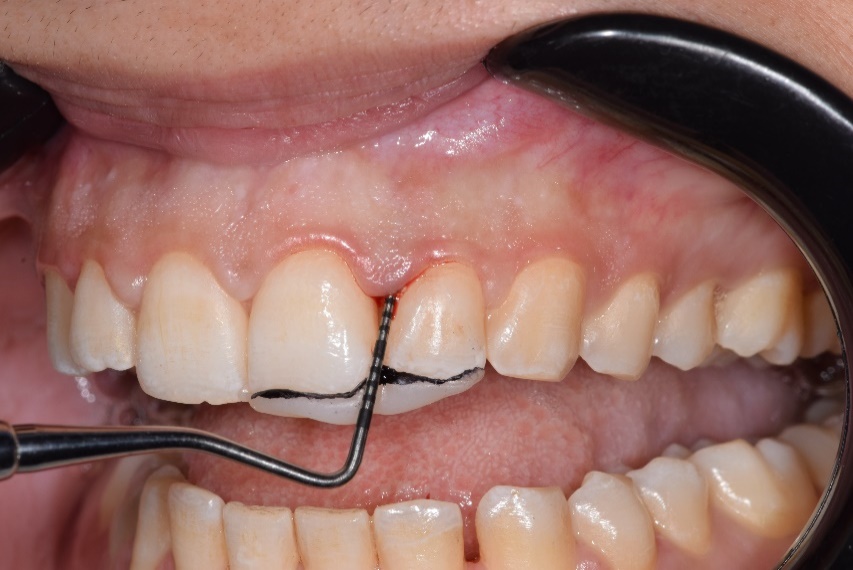
**

**Figure (S2): Composite stent used as a reference point for a standardized clinical measurement**


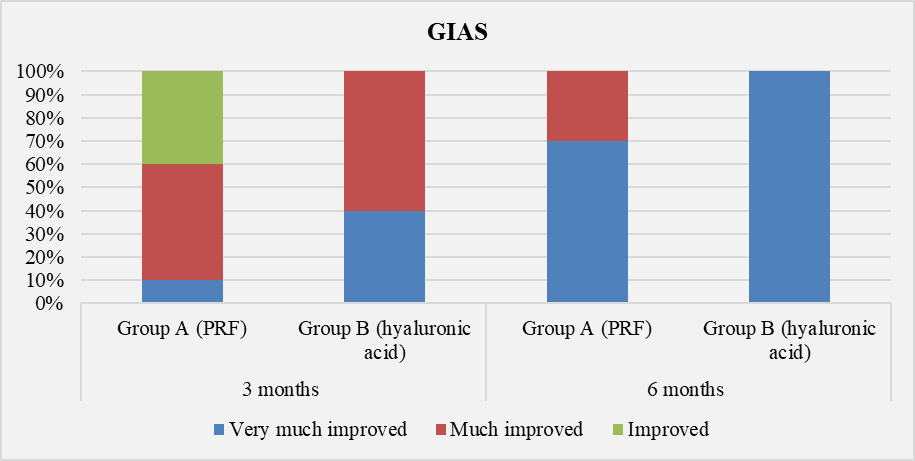


**Figure (S3): Frequency of GAIS scores in group A (L-PRF) and group B (HA)** **at baseline, 3 and 6 months**


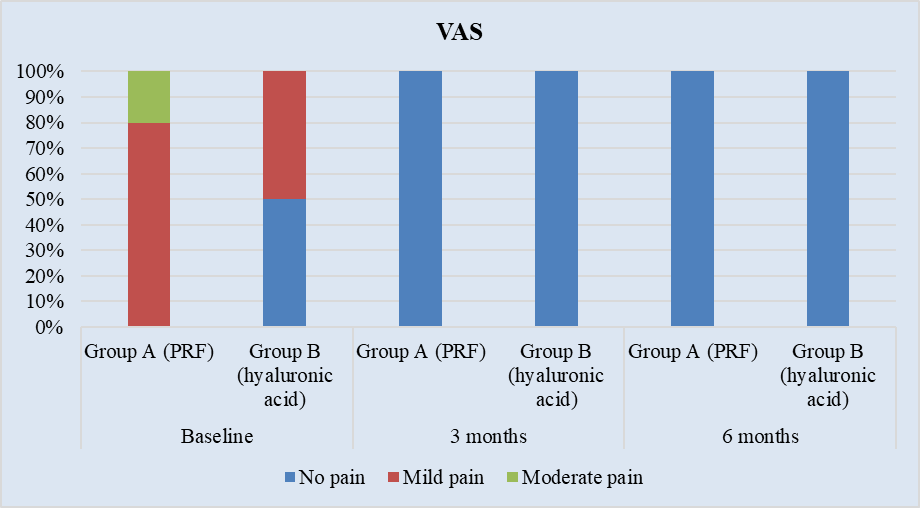


**Figure (S4): Frequency of VAS scores in (L-PRF) group and (HA) group at baseline, 3 and 6 months**

**
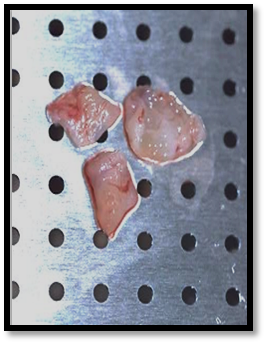
**

**Figure (S5): Cutting L-PRF membranes into multiple layers**


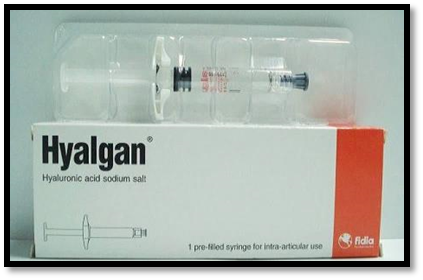


**Figure (S6): HYALGAN®, HA gel in pre-packed syringe**
